# Single‐trial EEG‐informed fMRI analysis of emotional decision problems in hot executive function

**DOI:** 10.1002/brb3.728

**Published:** 2017-05-29

**Authors:** Qian Guo, Tiantong Zhou, Wenjie Li, Li Dong, Suhong Wang, Ling Zou

**Affiliations:** ^1^ School of Information Science and Engineering Changzhou University Changzhou Jiangsu China; ^2^ Changzhou Key Laboratory of Biomedical Information Technology Changzhou Jiangsu China; ^3^ School of Life Science and Technology University of Electronic Science and Technology of China Chengdu Sichuan China; ^4^ Changzhou NO.1 People's Hospital affiliated with Suzhou University Changzhou Jiangsu China

**Keywords:** general linear model, hot executive function, K‐means clustering method, regressor, simultaneous EEG‐fMRI

## Abstract

**Background:**

Executive function refers to conscious control in psychological process which relates to thinking and action. Emotional decision is a part of hot executive function and contains emotion and logic elements. As a kind of important social adaptation ability, more and more attention has been paid in recent years.

**Objective:**

Gambling task can be well performed in the study of emotional decision. As fMRI researches focused on gambling task show not completely consistent brain activation regions, this study adopted EEG‐fMRI fusion technology to reveal brain neural activity related with feedback stimuli.

**Methods:**

In this study, an EEG‐informed fMRI analysis was applied to process simultaneous EEG‐fMRI data. First, relative power‐spectrum analysis and K‐means clustering method were performed separately to extract EEG‐fMRI features. Then, Generalized linear models were structured using fMRI data and using different EEG features as regressors.

**Results:**

The results showed that in the win versus loss stimuli, the activated regions almost covered the caudate, the ventral striatum (VS), the orbital frontal cortex (OFC), and the cingulate. Wide activation areas associated with reward and punishment were revealed by the EEG‐fMRI integration analysis than the conventional fMRI results, such as the posterior cingulate and the OFC. The VS and the medial prefrontal cortex (mPFC) were found when EEG power features were performed as regressors of GLM compared with results entering the amplitudes of feedback‐related negativity (FRN) as regressors. Furthermore, the brain region activation intensity was the strongest when theta‐band power was used as a regressor compared with the other two fusion results.

**Conclusions:**

The EEG‐based fMRI analysis can more accurately depict the whole‐brain activation map and analyze emotional decision problems.

## 1 INTRODUCTION

Executive function (EF) is a collection of top‐down processes which allows for conscious, goal‐directed control of thoughts and actions (Baptista, Osório, Costa Martins, Verissimo, & Martins, 2016). Functions including verbal reasoning, planning, sequencing, problem solving, the ability to sustain attention, and utilization of feedback are regarded as the “cold” components of executive functions as their corresponding cognitive processes are relatively “logical” or “mechanistic” based and tend to involve little emotional arousal. On the other hand, executive functions involving more “desires” or “emotion” such as the experience of reward and punishment, decision making with emotional and personal interpretation, and regulation of one's own social behavior are regarded as “hot” components (Blankenship, O'Neill, Deater‐Deckard, Diana, & Bell, 2016; Shields, Sazma, & Yonelinas, 2016). Researches have revealed that impairments of either “cold” or “hot” components could have devastating effects on people's daily activities, including the ability of work and study, function at home independently, and develop or maintain appropriate social relations (Ursache & Raver, 2015). In the studies of “hot” components of executive function, complementary evidence from scalp‐recorded event‐related potentials (ERPs) has shown that the “feedback negativity” (FN) is related to positive versus negative outcomes such as monetary rewards (Mensen et al., 2015); and variation in FN amplitude is considered to reflect the early evaluation of outcomes as either better or worse than expected (Lole, Gonsalvez, Barry, & De Blasio, 2013). In functional magnetic resonance imaging (fMRI) studies, the ventral striatum (VS) responds in anticipation of reward and other striatal areas including the caudate and the amygdala mediate the relationship between action and reward outcome (Tricomi, Delgado, & Fiez, 2004). Reward attainment and outcome monitoring recruit the medial prefrontal cortex (mPFC) (Iannaccone et al., 2015). A combined EEG and fMRI study reveals that for the win > loss comparison, fMRI activation in the mesocorticolimbic reward circuit including the VS and mPFC is positively correlated with FN (Carlson, Foti, Mujica‐Parodi, Harmon‐Jones, & Hajcak, 2011). However, the mechanism of “hot” executive function is not very clear at present. Gambling task can be well performed in the study of emotional decision. As fMRI researches focused on gambling task show not completely consistent brain activation regions (Happaney, Zelazo, & Stuss, 2004; Kerr & Zelazo, 2004; Li, Li, & D‘Argembeau, 2010), this study adopted EEG‐fMRI fusion technology to reveal brain neural activity related with feedback stimuli.

EEG has high temporal resolution for the underlying neuronal events but with low spatial resolution, whereas fMRI has high spatial resolution and low temporal resolution (Huster, Debener, Eichele, & Herrmann, 2012). The complementarity of EEG and fMRI data in time and space provides feasibility of the fusion research. There are currently three approaches to EEG/fMRI integration (Calhoun & Sui, 2016; Huster et al., 2012; Lei, Qiu, Peng, & Yao, 2010): (i) “symmetric fusion”, where a model is constructed to explain the EEG‐fMRI data. Symmetrical fusion does not assign a priori inferential preference to a given modality. The existing symmetrical fusion based on a cascade of generation models could provide a deeper understanding of the neural mechanisms underlying mental processes of interest. (ii) “Spatial constraint”, where spatial information from fMRI signals is used for source reconstruction of the EEG data. It generally applies independent component analysis (ICA) algorithm to extract regions of interest in brain function network from fMRI data. (iii) “Temporal prediction”, where the fMRI recordings are modeled with data from certain EEG signals obtained from ICA, such as P300 amplitude and alpha‐band power. EEG features can be convolved with a canonical hemodynamic response function (HRF) and the result of which can be used as a hemodynamic predictor in a general linear model (GLM). EEG/fMRI integration has been adopted in the studies of recognition memory (Hoppstädter, Baeuchl, Diener, Flor, & Meyer, 2015), attention modulation (Walz et al., 2014), spontaneous brain rhythms (Zhan et al., 2014), as well as epileptic discharges (Hunyadi et al., 2015), involving visual system (Walz et al., 2014), auditory system (Walz et al., 2015), and pain system (Christmann, Koeppe, Braus, Ruf, & Flor, 2007). Therefore, EEG‐fMRI fusion analysis may be beneficial in understanding the mechanism of “hot” executive function.

In this work, we applied an EEG‐informed fMRI analysis to process simultaneous EEG‐fMRI data during the monetary gambling task, which is most widely adopted in the study of emotional decision concerning “hot” components of executive function. EEG trial‐by‐trial amplitudes of the feedback‐related negativity (FRN) and the powers of alpha‐ and theta‐band related to feedback were separately used as regressors in general linear models (GLMs), and the fusion results with different regressors were compared.

## 2 MATERIALS AND METHODS

### 2.1 Participants

Twenty healthy, right‐handed participants (17 men and 3 women aged from 19 to 25, the mean age was 23, the standard deviation was 1.48) were involved in this study. Participants had normal or corrected normal vision without a history of neurological, medical, or psychiatric disorders. All participants provided written informed consent to be part of the experiment, and the study was approved by the local ethics committee (Changzhou University, Changzhou, China).

### 2.2 Task procedure

**Figure 1:**
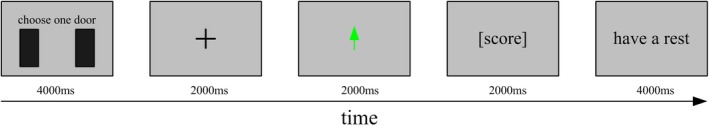
Experimental paradigm of gambling task

All participants performed the monetary gambling task (Carlson et al., 2011). Each trial began with two doors presented side‐by‐side on the screen for 4,000 ms. Participants were instructed that behind one of the doors there was a monetary prize (+ ¥2.0), whereas behind the other door there was a loss (− ¥1.0). A MRI‐compatible response box was used to make the choice of door. In addition, participants were told that if they did not choose when the doors were on the screen, the computer would choose randomly. Next, after a brief fixation cue (2,000 ms), a feedback screen was displayed (2,000 ms) where a green “↑” indicated a correct guess, whereas a red “↓” indicated an incorrect guess. Then, the total scores that the participant had obtained so far were presented in the center of the screen (2,000 ms) and the interval between each trial was 4,000 ms (Figure 1). The task was 18 min and 40 s in duration and consisted of 80 trials over eight individual scans with wins and losses presented in random order. Experiments were performed by e‐prime software and this software recorded behavioral data at the same time. The mean reaction time (the latency to choose a door) was computed separately on trials.

### 2.3 Simultaneous EEG‐fMRI data recording

The subjects were scanned on a 3‐T scanner (Philips Medical Systems) while wearing an EEG‐Cap (HydroCel Geodesic Sensor Net; Electrical Geodesics, Inc., Eugene, OR). Functional MRI images were acquired in a BOLD‐sensitized EPI T2*‐weighted sequence with a repetition time of 2,000 ms (echo time of 35 ms and flip angle of 90^o^). Twenty‐four continuous slices parallel to the anterior commissure‐posterior commissure line were acquired per volume (field of view of 230 × 182 mm and matrix of 96 × 74). A T1‐weighted structural image (1 × 1 × 1 mm) was also acquired for each participant in the experiment.

EEG recordings were acquired with a sampling rate of 250 Hz with Net Station EEG Software (RRID:nlx_155825, Electrical Geodesics Inc.), using 64 channels in 10–10 montage. In the data collecting period, the impedance of all electrodes was kept below 50 kΩ and all electrodes were referenced to a point at infinity. Clock synchronization box ensured the simultaneous EEG‐fMRI data recording.

### 2.4 EEG data processing

The collected EEG data were preprocessed to remove the artifacts with the Net Station Software, such as gradient field noise, cardiac artifacts, and power‐frequency interference. First, the raw EEG data were corrected for the gradient artifacts using average artifact subtraction (AAS) algorithm and cardiac artifacts were suppressed by optimal basis set (OBS) algorithm. Next, the FIR filter with the pass‐band of 0.1 Hz to 30 Hz was applied. The data were segmented for each trial, beginning 200 ms before feedback onset and continuous for 1,000 ms following feedback onset, and was performed a baseline correction from −200 ms to 0 ms. Then, artifact detection and bad channel replacement were applied to each channel and segment. The Reference Electrode Standardization Technique was applied to standardize the reference of scalp EEG recordings to a point at infinity that, being far from all possible neural sources, acts like a neutral virtual reference (Yao, 2001).

As there were some electromyography, eye movements, and other noise contained in the EEG data after preprocessing, ICA algorithm was adopted to remove the noise components. ICA is widely used in the blind source separation algorithm and the logistic infomax ICA was performed in this study. Researches utilizing ERP indicate that FRN is sensitive to win versus loss feedback as well as outcome expectation and normally appears during 200‐350 ms following feedback onset in FCz electrode (Thoma, Edel, Suchan, & Bellebaum, 2015). Therefore, the magnitude of FRN in each single trail was convolved with the canonical HRF and then performed as a regressor in GLM. Meanwhile, recent studies have shown that there are differences in alpha‐band and theta‐band power under different feedback conditions and relative EEG power which approximated neglecting insignificant frequency bands characterizes the experimental task activity better than the absolute power (Abouzari, Oberg, & Tata, 2016). So, the relative alpha‐band and theta‐band power of each trial over FCz electrode during 200‐350 ms time period after feedback onset was also extracted as regressors.

### 2.5 fMRI data processing

The fMRI data were preprocessed and analyzed with the SPM8 software (http://www.fil.ion.ucl.ac.uk/spm/, RRID:SCR_007037). First, the fMRI images were corrected for slice‐timing artifacts and spatially realigned to the first brain volume. The results were normalized based on the Montreal Neurologic Institute (MNI) reference brain and the voxel sizes were turned into 3 × 3 × 3 mm. Then fMRI maps were smoothed by an 8‐mm FWHM Gaussian kernel. As the BOLD signal always delay 4‐8 s, therefore, five time points after stimulus presentation were taken in each trial. After *z*‐score processing, k‐means clustering method was performed to make a mask and remove some of the data unrelated to the research background based on this mask. K‐means clustering is based on hard divided guidelines and defines each object that can only be segregated into one class. The main idea is to calculate cluster centers by performing multiple iterations following the principle of higher similarity in cluster and lower similarity intercluster. The similarity of a cluster can be defined by the average value. Finally, 90 regions of interest (ROIs) were extracted based on AAL template and the mean activation intensity of each ROI was calculated, respectively.

### 2.6 EEG‐informed fMRI analysis

**Figure 2:**
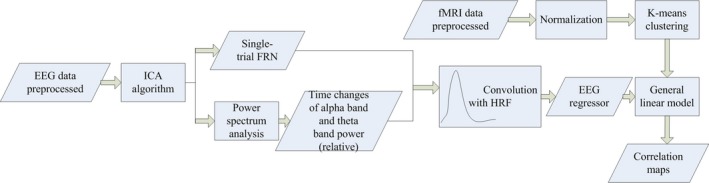
Block scheme of EEG‐based fMRI analysis

The entire process is shown in a block scheme in Figure 2. EEG‐informed fMRI analysis applied EEG signals as predictor variables to model fMRI time processing. Based on the linear coupling assumption of neurovascular, the predicted BOLD signals were constituted of the extracted EEG features convolved by standard HRF and were used to find the relevant activation areas reflecting neural activity in the whole‐brain BOLD signals.

**Figure 3:**
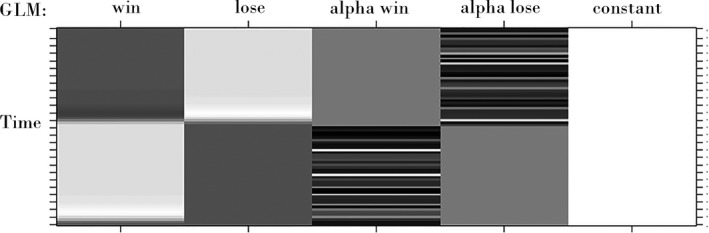
Example of design matrix for joint first‐level EEG‐fMRI analysis

In order to obtain correlation maps, EEG‐based regressors and fMRI data after clustering were put into GLMs. In this study, a complete EEG‐fMRI integration analysis was composed of two separate computing phases. During the first phase, each EEG regressor of each subject was performed as a multiple linear regression in GLM separately and the relationship between fMRI data and the EEG regressor was calculated according to Eq. (1).

(1) $$Y=[X_{1},X_{2},X_{3}]*\beta +\varepsilon$$

where *Y* represents the processed fMRI (BOLD) data, *X*_*1*_, *X*_*2*_, and *X*_*3*_ represent the model matrices separately containing regressors of EEG trial‐by‐trial amplitudes of FRN and the power of alpha‐ and theta‐band related with feedback, in other words the model signals searched in *Y*. The values in matrix β describe how much a given model signal affects the variability in the BOLD signal in a specific voxel. Matrix ε is the residual variability in the data. A typical model matrix represented by *X*_*3*_ in equation (1) is shown in Figure 3. It contains fixed amplitude of 1 as regressors for win and lose stimulus, theta‐band power‐based regressors, as well as constant terms in BOLD signals.

As three EEG regressors were calculated for 20 subjects, it means that 60 (20 subjects × 3 GLMs) separate GLM estimations were performed. As the analysis only focused on their mutual influences, one SPM showed 3D correlation map between local BOLD signal and EEG regressors for corresponding subject.

During the second phase, group analyses were performed with SPMs of subjects via a single‐sample *t* test on contrasts of the EEG‐related regressors in both win and loss conditions. All significant voxels were identified by a voxel‐level threshold *P*_*uncorrected*_ **< .001.

## 3 RESULTS

Based on the recording data of 20 participants, the behavioral responses were statistically analyzed. The type of feedback that participants received on the previous trial had a significant effect on the latency to make a choice (*p* = .008). Following win stimuli participants took more time to choose a door than following loss stimuli (following win stimuli: *M* = 1,149 ms, *SD* = 142 ms; following loss stimuli: *M* = 875 ms, *SD* = 86 ms).

**Figure 4:**
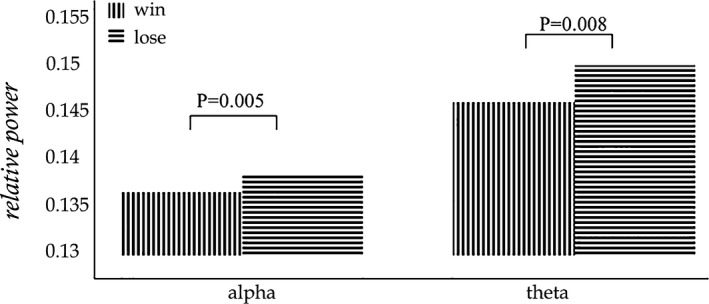
Mean relative powers of alpha band and theta band over FCz electrode

**Figure 5:**
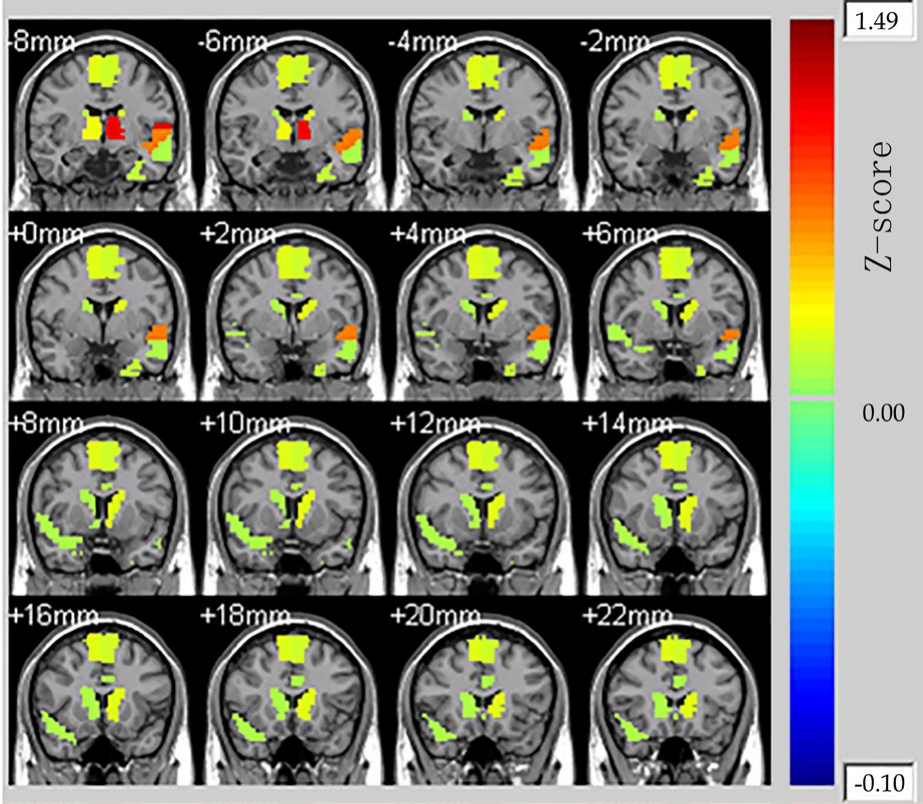
Whole‐brain activation map by fMRI analysis for monetary wins compared to losses

The mean relative powers of alpha band and theta band were shown in Figure 4. The power of theta band was greater than that of alpha band, and in these two interest‐frequency bands the power under lose stimuli was all significantly higher than that under win stimuli. The *p* value between win and lose stimuli in alpha‐band power was 0.005 and in theta‐band power was 0.008 came from Independent sample *t* test. Thus, the alpha‐ and theta‐band relative power could be used as regressors of GLMs. The whole‐brain fMRI analysis revealed significant activations in Cingulum_Ant_L, Cingulum_Ant_R, Caudate_L, and Caudate_R in the Automated Anatomical Labeling (AAL) template. The results could be seen in Figure 5 which display 16 different brain slices. The colored parts were the activation areas and the color plate on the right showed the value of *Z*‐score which represented the activation intensity. All clusters were with an extent threshold of 10 continuous voxels. Table 1 provides a detailed statistical description of the above‐mentioned activation regions as well as other areas related to reward processing. AAL template and its corresponding coordinate are provided by Montreal Neurological Institute.

**Table 1:** Reward‐ versus loss‐related activations by fMRI analysis

Brain region		AAL Lable	Centers[MNI]			*z*‐score (win vs. lose)
			x (mm)	y (mm)	z (mm)	
Cingulum_Ant_L	Anterior cingulate and paracingulate gyri	31	−4.04	35.4	13.95	0.12
Caudate_L	Caudate nucleus	71	−11.46	11	9.24	0.24
Caudate_R	Caudate nucleus	72	14.84	12.07	9.42	0.13

AAL, anatomical labeling template; MNI, montreal neurologic institute.

**Figure 6:**
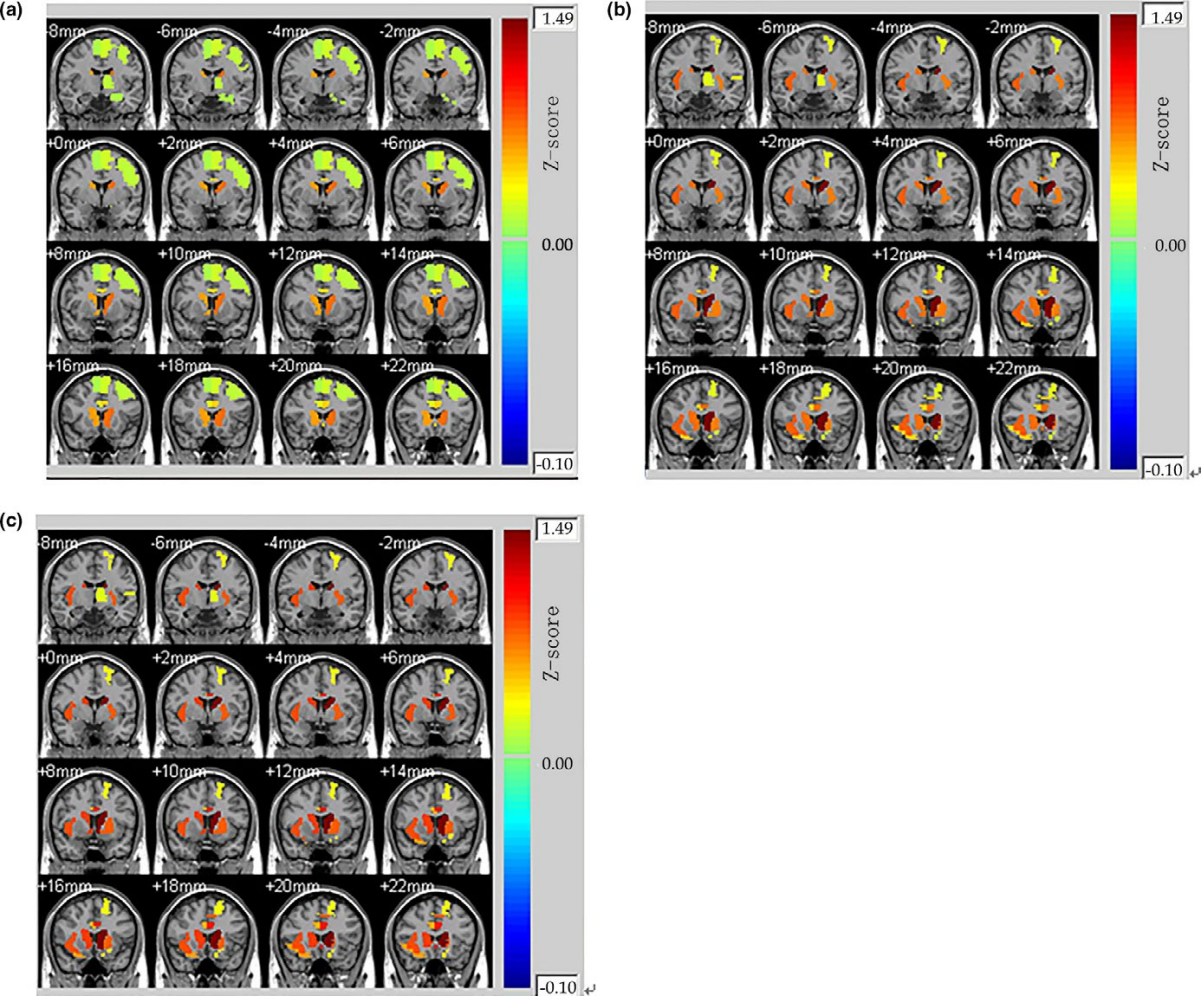
Whole‐brain activation map by GLM analysis a) when FRN amplitude performed as a regressor, b) when alpha‐band power performed as a regressor, c) when theta‐band power performed as a regressor for monetary wins compared to losses

To identify brain areas showing BOLD responses correlating with FRN amplitudes, alpha‐band, and theta‐band power, an EEG–fMRI integration‐by‐prediction analysis was conducted, with single‐trial EEG feature estimates performed as additional regressors in the SPM GLM analysis. Group analysis was then carried out for the win versus loss contrast to assess the brain regions involved in reward processing. Caudate_L, Caudate_R, Cingulum_Post_L, Cingulum_Post_R, Frontal_Mid_Orb_L, and Hippocampus_L showed significant activation when amplitude of FRN was performed as a regressor in GLM (Figure 6a and Table 2). The subjective feeling of hedonia is associated with OFC activation, so the OFC activated as expected for the win > loss comparison. The activation in the posterior cingulate gyrus and the hippocampus was associated with emotion of participants after feedback onsets. Furthermore, the activation intensity of the caudate was stronger compared with the only fMRI analysis. The subcortical activation regions when alpha‐band power entered as a regressor in GLM analysis were most similar with the activation areas when theta‐band power was used as a regressor (Figure 6b and Figure 6c). For win stimuli compared with lose stimuli, we found significantly greater activation in Frontal_Sup_Orb_L, Frontal_Mid_Orb_L, Frontal_Inf_Orb_R, and Frontal_Sup_Medial_L. The results of these two analyses showed significantly greater activation in the left middle frontal gyrus compared with FRN‐based results. In addition, there was also a large amount of activation left in the lenticular nucleus which is included in the VS. As reflected in the Figure 6b, Figure 6c, and Table 3, this area presented greater activation in comparison with the most other activated regions. Bilaterally in the caudate, anterior cingulate, and paracingulate gyri showed stronger activation intensity when compared with either conventional fMRI analysis or fusion analysis based on FRN regressors. Although the EEG‐informed fMRI results based on alpha‐band power and the results based on theta‐band power revealed the same activation regions, the intensity of all extracted reward‐related activation areas of the theta‐band power was stronger than the results of the alpha‐band power (Table 3). The *p* value of the two intensity results is 0.002 came from paired *t* test. It shows that the two results have significant differences.

**Table 2:** Reward‐ versus Loss‐related activations by GLM analysis when FRN amplitude performed as a regressor

Brain region		AAL Lable	Centers[MNI]			*z*‐score (win vs. lose)
			x (mm)	y (mm)	z (mm)	
Frontal_Mid_Orb_L	Middle frontal gyrus, orbital part	9	−30.65	50.43	−9.62	0.21
Cingulum_Ant_L	Anterior cingulate and paracingulate gyri	31	−4.04	35.4	13.95	0.51
Cingulum_Ant_R	Anterior cingulate and paracingulate gyri	32	8.46	37.01	15.84	0.26
Cingulum_Post_L	Posterior cingulate gyrus	35	−4.85	−42.92	24.67	0.32
Cingulum_Post_R	Posterior cingulate gyrus	36	7.44	−41.81	21.87	0.25
Hippocampus_L	Hippocampus	37	−25.03	−20.74	−10.13	0.11
Caudate_L	Caudate nucleus	71	−11.46	11	9.24	0.79
Caudate_R	Caudate nucleus	72	14.84	12.07	9.42	0.62

GLM, general linear models; AAL, anatomical labeling template; MNI, montreal neurologic institute.

**Table 3:** Reward‐ versus loss‐related activations by GLM analysis when power features performed as regressors

Brain region		AAL Lable	Centers[MNI]			*z*‐score (win vs. lose)	
			x (mm)	y (mm)	z (mm)	alpha‐band power	theta‐band power
Frontal_Sup_Orb_L	Superior frontal gyrus, orbital part	5	−16.56	47.32	−13.31	0.20	0.38
Frontal_Mid_Orb_L	Middle frontal gyrus, orbital part	9	−30.65	50.43	−9.62	0.32	0.54
Frontal_Inf_Orb_R	Inferior frontal gyrus, orbital part	16	41.22	32.23	−11.91	0.49	0.65
Frontal_Sup_Medial_L	Superior frontal gyrus, medial	23	−4.8	49.17	30.89	0.51	0.82
Frontal_Sup_Medial_R	Superior frontal gyrus, medial	24	9.1	50.84	30.22	0.34	0.42
Cingulum_Ant_L	Anterior cingulate and paracingulate gyri	31	−4.04	35.4	13.95	0.79	1.02
Cingulum_Ant_R	Anterior cingulate and paracingulate gyri	32	8.46	37.01	15.84	0.47	0.58
Caudate_L	Caudate nucleus	71	−11.46	11	9.24	1.49	1.72
Caudate_R	Caudate nucleus	72	14.84	12.07	9.42	0.79	0.92
Putamen_L	Lenticular nucleus, putamen	73	−23.91	3.86	2.4	0.74	0.81

GLM, general linear models; AAL, anatomical labeling template; MNI, montreal neurologic institute.

## 4 DISCUSSION AND CONCLUSION

Emotional decision is an important part of “hot” executive function and gambling task is probably the most frequently applied task in the study of emotional decision. Completing gambling task requires cognitive abilities of working memory and inhibitory control as well as emotion and social abilities. Sergeant (Sergean, Geurts, Huijbregts, Scheres, & Oosterlaan, 2003) proposed a cognitive‐energetic model and the effects of reward and punishment have been associated in the cognitive‐energetic model as being critical to the operation of the effort pool. The energetic component of that model might be considered to be a “bottom‐up” system which registers and gives feedback to the orbital frontal cortex on whether a particular stimulus–response relation is satisfying or aversive for the organism. Initial studies with the Iowa Gambling Task (IGT) found it to be sensitive to ventromedial (VM) and OFC functioning, as patients with lesions in these brain regions exhibited deficient IGT scores and difficulties in real‐world decision making despite normal range performance on other neuropsychological tests (Bechara, Damasio, Damasio, & Lee, 1999). Subsequent functional imaging research has confirmed a connection between IGT performance and VM/OFC function (Li, Zhong‐Lin, D'Argembeau, Ng, & Bechara, 2010). Several studies observed significantly more risky decision‐making patterns in Parkinson's disease (PD) patients than in healthy controls (Xi et al., 2015). Those potential impairments have been explained by dysfunctional activity in the frontostriatal network and, more precisely, the orbitofrontal–ventrostriatal pathway (Michele & Ubaldo, 2012) which is considered to regulate motivational, that is, reward‐related processes.

As the whole‐brain activation maps are different under win and lose stimuli in gambling task, EEG‐informed fMRI analysis has significant meaning in analyzing emotional decisions. The mesocorticolimbic dopamine (DA) system, including dopaminergic projections from the ventral tegmental area to both the VS/nucleus accumbens and dorsal striatum (i.e., caudate and putamen) as well as OFC, mPFC, and amygdala, has long been implicated in reward processing (Carlson et al., 2011). In this study, EEG‐based fMRI analysis under FRN, alpha‐band, and theta‐band power were performed separately and the fusion analysis all extracted more reward‐related brain regions representing reactions of participants to reward and punishment stimulation than the conventional fMRI results. Behavior results also show different reaction time caused by feedback. Among the three additional regressors, the results based on the theta‐band power revealed more brain areas relevant to reward processing such as the caudate, the OFC, as well as the VS, and the intensity of these activation areas were stronger than the other two analyses. Cingulum cortex is considered to be closely linked to emotional memory and caudate could mediate the relationship between action and reward outcome. Meanwhile, caudate and the VS play an important role in the mesocorticolimbic reward circuit. Based on the calculation of relative EEG power and the fMRI feature extraction with k‐means clustering algorithm, GLM was performed to obtain whole‐brain activation maps using joint EEG‐fMRI data in the gambling task in this work. EEG features were used as predictor variables to model fMRI time processing and based on the linear coupling assumption of neurovascular, the activation results combined with EEG signals depicted the whole‐brain activation map more reasonably. Most fMRI researches indicate that the OFC is mainly activated in gambling task (Happaney et al., 2004; Kerr & Zelazo, 2004). There are also some fMRI researches showing that the main activation region is dorsolateral prefrontal cortex (Li et al., 2010). The literature results at home and abroad are not completely consistent. Results in this study were consistent with some previous literature reports (Carlson et al., 2011; Li et al., 2010) and confirmed that components of the mesocorticolimbic DA system mediate reward processing from seeking to gratification.

Several limitations should be considered in this work. First, simultaneous EEG‐fMRI is a noninvasive technique with both high temporal and spatial resolution, and same stimulus and states of subjects are ensured by the simultaneous recording. However, large artifact in EEG data obtained from the MRI scanner would cause lower signal‐to‐noise ratio (Lei, Valdes‐Sosa, & Yao, 2012). Sampling rate of 250 Hz used in the study is low, so extraction of FRN component may not be accurate. EEG sampling rate of 1,000 Hz or higher would be set in later experiments. In addition, longer experimental time results from wearing electrode cap as well as other preparation steps would bring discomfort to participants and affect the quality of advanced cognitive tasks. Second, the limitation of K‐means clustering is that the cluster number k needs to be determined in advance. It generally requires a large number of tests and the clustering results are sensitivity to this parameter (Yedla, Rao Pathakota, & Srinivasa, 2010). Third, a canonical HRF was applied across all EEG regressors. In recent years, it has been revealed with different analysis methods that although the amplitude time delay is consistent, the impulse response function (IRF) between EEG and BOLD signal differs with respect to frequency band of interest (Bridwell, Lei, Eichele, & Calhoun, 2013). In the future work, it might be more meaningful to calculate with different IRFs or to deconvolve IRFs between EEG regressors and BOLD signal. Moreover, we will analyze other modal data (EDA and ECG) at the same time in further studies to study emotional decision problems deeply.

In conclusion, the EEG‐informed fMRI analysis based on single‐trial EEG amplitude and rhythm characteristics could precisely extract the activation brain regions relevant to reward processing and provide a good research method for the subsequent analysis of emotional decisions. The study of “hot” executive function has important guiding significance of the development of our cognition as well as affection and can also provide theoretical bases for the treatment of ADHD, autism, and other disorders.
